# Tracheobronchopathia osteochondroplastica: rare but not to be forgotten

**DOI:** 10.1002/rcr2.609

**Published:** 2020-06-26

**Authors:** Fahad Gul, Eric Peterson, Robert Dejoy, Glenn Eiger, Andres Mora Carpio, Ena Gupta

**Affiliations:** ^1^ Department of Internal Medicine Einstein Medical Center Philadelphia PA USA; ^2^ Department of Pulmonary and Critical Care Einstein Medical Center Philadelphia PA USA

**Keywords:** Bronchoscopy, ossification, tracheal disease, tracheobronchopathia osteochondroplastica

## Abstract

We report a case of a 59‐year‐old male with a one‐month history of pleuritic chest pain who was diagnosed with tracheobronchopathia osteochondroplastica (TO). TO is a rare benign condition characterized by protruding submucosal nodules into the tracheobronchial lumen. The disease is generally asymptomatic, with rare cases of progressive nodularity, cough, dyspnoea, and haemoptysis. Diagnosis can be made via bronchoscopic visualization of bony and cartilaginous nodules on tracheal walls. Although generally benign, the rarity of this condition makes diagnosis difficult even for trained pulmonologists and frequently predisposes patients to unnecessary invasive diagnostic testing and improper management of symptoms and contributing co‐morbid conditions. We present this case to increase physician and patient awareness about this disease to help improve diagnostic strategy and knowledge of disease manifestations and potential complications.

## Graphical abstract

We report a case of a 59‐year‐old male with a one‐month history of pleuritic chest pain who was diagnosed with tracheobronchopathia osteochondroplastica (TO). TO is a rare benign condition characterized by protruding submucosal nodules into the tracheobronchial lumen. We hope this case will help to increase physician and patient awareness about this disease to help improve diagnostic strategy and knowledge of disease manifestations and potential complications.

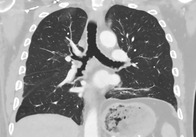

## Introduction

Tracheobronchopathia osteochondroplastica (TO) is a rare, idiopathic, non‐neoplastic disease characterized by protrusion of diffuse cartilaginous and osseous nodules within the tracheobronchial lumen [1]. The majority of patients are asymptomatic and those with symptoms usually present with variable non‐specific symptoms. Historically, diagnosis has largely been made post‐mortem; however, the development of bronchoscopy and improvement in computed tomography (CT) imaging quality have allowed for increased recognition [2]. Lack of knowledge and understanding of the disease frequently results in unnecessary invasive diagnostic intervention and misdiagnosis with subsequent mismanagement.

## Case Report

**Figure 1:**
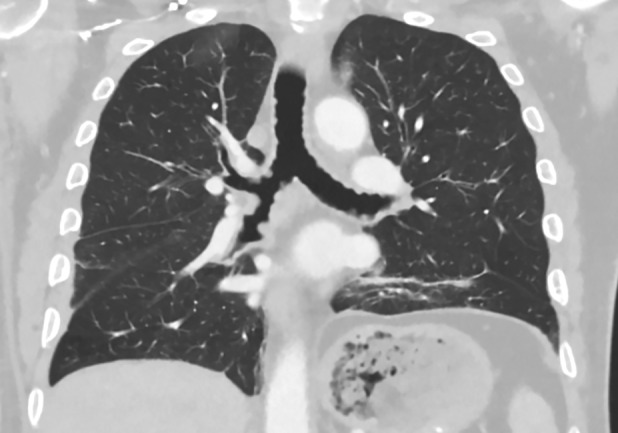
Coronal section of computed tomography (CT) of the chest (lung windows) showing multiple protruding nodularities in the tracheal bronchial lumen.

**Figure 2:**
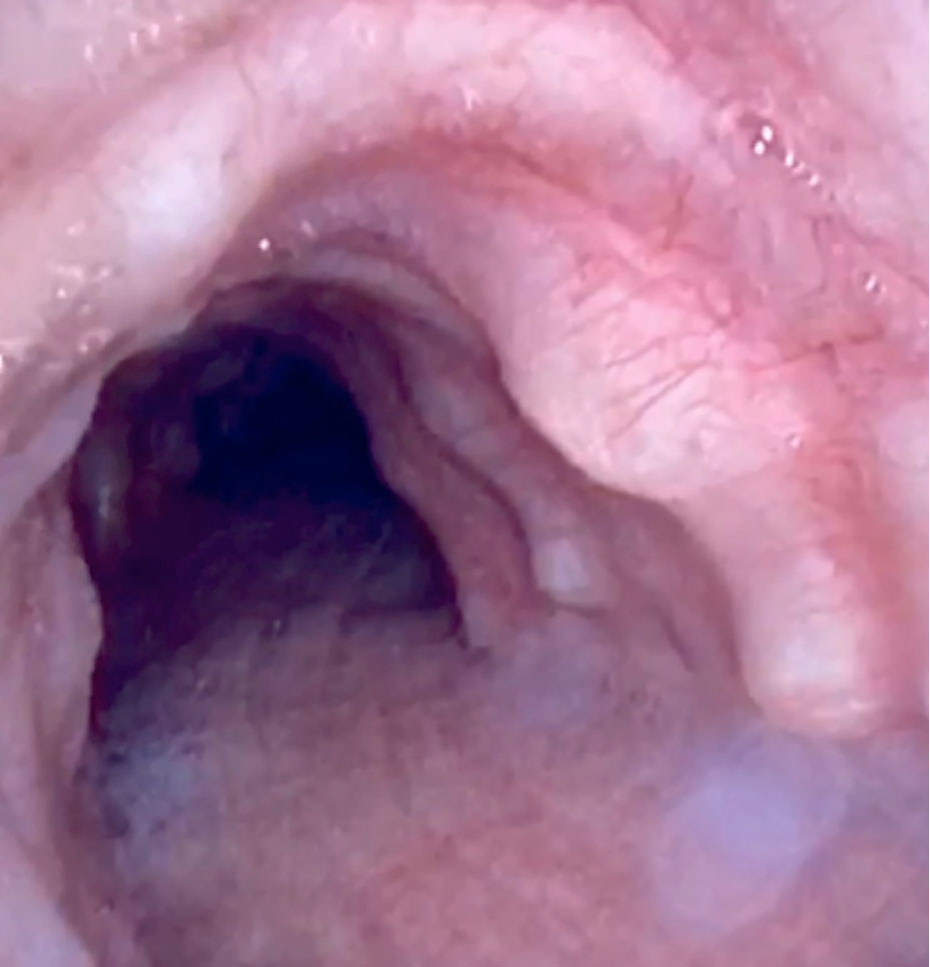
Bronchoscopic view of the trachea showing mucosal nodules anteriorly and laterally with sparing of the posterior wall of the trachea.

A 59‐year‐old Caucasian male presented with unstable angina requiring percutaneous coronary intervention (PCI). Acute chest pain improved post‐procedure; however, he continued to report a one‐month history of persistent right‐sided pleuritic chest pain and worsening dyspnoea. CT of the thorax demonstrated diffuse tracheal wall nodularity (Fig. 1). In addition, consolidation of the posterior segment of the right lower lobe (RLL) was visualized with adjacent liner opacities suspicious for pneumonia with a small right‐sided pleural effusion. He had no associated fevers, chills, haemoptysis, or weight loss. He denied any known history of allergies, lung disease, or recurrent respiratory tract infections. Laboratory work was unremarkable for leucocytosis with normal liver and kidney function tests. Flexible bronchoscopy was performed to evaluate for infectious versus neoplastic process. Tracheal visualization revealed multiple sessile nodules protruding into the tracheobronchial lumen extending to the proximal bronchi with sparing of the posterior membranous wall of the trachea (Fig. 2). RLL bronchus and segmental bronchi were patent without intraluminal abnormalities. Bronchoalveolar lavage (BAL) was performed and cultures grew *Haemophilus influenzae*. Decision was made to withhold biopsy due to elevated bleeding risk from dual antiplatelet therapy initiation as a result of recent PCI. Right‐sided thoracentesis was performed with removal of 250 mL of neutrophilic exudative yellow cloudy fluid. Pleural fluid cultures were negative and cytology was negative for malignant cells. The patient symptomatically improved with resolution of pleuritic chest pain. He was discharged with short‐course antibiotics and education regarding bronchoscopy findings and possible future implications.

## Discussion

TO is generally a rare benign disease characterized by the presence of multiple cartilaginous and osseous structures protruding into the trachea and larger bronchi. Incidence rates of TO as reported by Meyer et al. are three in 1000 (0.3%) autopsies and between one in 125 (0.8%) and one in 5000 (0.02%) bronchoscopies [3]. The disease has a slight predilection towards males with a mean age of diagnosis at 52 years, although cases young as 9 years have been described [4, 5].

Clinical symptoms of TO are variable and non‐specific including chronic cough, dyspnoea on exertion, and wheezing which can be misdiagnosed as asthma. Symptoms can correlate with site and degree of obstruction, with larger lesions predisposing to airway obstruction and increased risk of recurrent infection, bronchiectasis, and nodule ulceration [6]. The aetiology remains largely unclear. Histopathological examination of bronchoscopic specimens has found infiltration of inflammatory and cartilaginous cells causing metaplastic epithelial changes [7]. This architectural distortion with alteration of the mucosal surface and reduced mucociliary clearance can predispose to recurrent infections [8]. Even though pathogenesis is uncertain, chronic inflammation may have a role in affecting symptoms and progression of disease. A retrospective study of 41 patients reported a progression rate of 45% [9]. Our patient presented with an RLL pneumonia and parapneumonic effusion. Alteration in host defence mechanisms due to could have predisposed this patient to his first case of pneumonia even without focal airway obstruction. He has no obvious exposures or risk factors for lower respiratory tract infection. We discussed with him airway hygiene and his risk factors for recurrent infection and prevention modalities.

In our patient, TO was suspected based on CT findings. The diagnosis was confirmed via bronchoscopic visualization of protruding osseous structures into the tracheobronchial lumen with sparing of the posterior membranous wall. The posterior tracheal wall is composed of fibromuscular tissue without a cartilaginous component capable of developing osseous nodules. This feature is helpful in differentiation from non‐localizing pathologies including tracheal bronchial amyloidosis, granulomatosis with polyangiitis, and sarcoidosis [10, 11, 12, 13]. CT imaging is an effective non‐invasive tool to identify irregular and calcified nodular lesions in the trachea; however, it does not establish diagnosis, as other disease processes such as relapsing polychondritis may appear similar radiographically [10]. Bronchoscopic visualization of characteristic anterolateral lesions remains the gold standard for diagnosis; however, decision on biopsy for confirmation remains controversial and is reasonable to pursue in patients with symptomatic disease, those planned for endobronchial intervention, and when alternative diagnosis is being considered [14]. In our case, we performed bronchoscopy for diagnostic confirmation and evaluation of opacity and made a decision to withhold biopsy given characteristic bronchoscopic TO features and increased risk for procedural‐related complications given recent PCI.

Patients affected by TO generally do not require treatment, given that the majority of patients are asymptomatic. Rigid bronchoscopy under general anaesthesia has been shown to be successful in debulking large obstructive lesions that would otherwise be labour‐intensive, inadequate, and have higher risk of complication with flexible bronchoscopy [15]. Recurrent infections and atelectasis are treated through conventional methods unless caused by focal obstruction. There are reports suggesting improvement in symptoms with administration of inhaled steroids and chest physiotherapy [16]. In our case, we treated our patient with an antibiotic course for his pneumonia complicated by parapneumonic effusion. We further provided education to the patient regarding the potential for progression of his condition and awareness of potential complications during anaesthesia and intubation [17]. We further counselled on the importance of avoiding inflammatory insults including tobacco smoke, toxic inhalations, and adequate control of acid reflux and asthma. More studies are needed to determine which patients have the highest risk of progression and what preventative measures can be undertaken to reduce the risk of progression and subsequent disease manifestations.

TO is a rare, typically benign disease with a variable clinical presentation and prognosis. Diagnosis is established with bronchoscopic visualization without the need for biopsy in most cases. Treatment is supportive and treatment of chronic inflammatory conditions is key. Early identification is important to prevent disease complications and progression.

### Disclosure Statement

Appropriate written informed consent was obtained for publication of this case report and accompanying images.
